# Engineering of a modular and synthetic phosphoketolase pathway for photosynthetic production of acetone from CO_2_ in *Synechococcus elongatus* PCC 7942 under light and aerobic condition

**DOI:** 10.1111/pbi.12536

**Published:** 2016-02-16

**Authors:** Jun‐Won Chwa, Wook Jin Kim, Sang Jun Sim, Youngsoon Um, Han Min Woo

**Affiliations:** ^1^ Clean Energy Research CenterKorea Institute of Science and TechnologySeongbuk‐guSeoulKorea; ^2^ Green School (Graduate School of Energy and Environment)Korea UniversitySeongbuk‐guSeoulKorea; ^3^ Department of Chemical and Biological EngineeringKorea UniversitySeongbuk‐guSeoulKorea; ^4^ Department of Clean Energy and Chemical EngineeringKorea University of Science and TechnologyYuseong‐guDaejeonKorea

**Keywords:** *Synechococcus elongatus *PCC 7942, metabolic engineering, acetone, biosolar cell factories

## Abstract

Capture and conversion of CO_2_ to valuable chemicals is intended to answer global challenges on environmental issues, climate change and energy security. Engineered cyanobacteria have been enabled to produce industry‐relevant chemicals from CO_2_. However, the final products from cyanobacteria have often been mixed with fermented metabolites during dark fermentation. In this study, our engineering of *Synechococcus elongatus* PCC 7942 enabled continuous conversion of CO_2_ to volatile acetone as sole product. This process occurred during lighted, aerobic culture via both ATP‐driven malonyl‐CoA synthesis pathway and heterologous phosphoketolase (PHK)‐phosphotransacetylase (Pta) pathway. Because of strong correlations between the metabolic pathways of acetate and acetone, supplying the acetyl‐CoA directly from CO_2_ in the engineered strain, led to sole production of acetone (22.48 mg/L ± 1.00) without changing nutritional constraints, and without an anaerobic shift. Our engineered *S. elongatus* strains, designed for acetone production, could be modified to create biosolar cell factories for sustainable photosynthetic production of acetyl‐CoA‐derived biochemicals.

## Introduction

Concerns about energy security, and environmental issues affecting the sustainable carbon cycle, has focused attention on engineering photosynthetic organisms including plants that might sequester and convert CO_2_ to organic materials using solar energy. Genetically modified cyanobacteria have been enabled to produce short‐chain alcohols such as ethanol (Deng and Coleman, 1999), isopropanol (Hirokawa *et al*., 2015; Kusakabe *et al*., 2013), isobutanol (Atsumi *et al*., 2009) and 1‐butanol (Lan and Liao, 2012; Lan *et al*., 2013); biochemicals such as ethylene (Ungerer *et al*., 2012; Xiong *et al*., 2015), 2‐methyl‐1‐butanol (Shen and Liao, 2012) and 2,3‐butanediol (Oliver *et al*., 2013); and isoprenoids such as limonene (Davies *et al*., 2014; Kiyota *et al*., 2014) and α‐bisabolene (Davies *et al*., 2014) by manipulating their metabolic pathways and introducing heterologous pathways. Conversion of CO_2_ to biochemicals using cyanobacteria requires production under light and dark conditions. However, neither continuous photosynthetic production of such biochemicals, nor the processes by which they might be separated, has yet been studied extensively.

The nature of the target chemicals is important for the continuous conversion of CO_2_. Highly volatile isoprene is a desirable target chemical because of the instant separation possible during photosynthetic cell growth. Engineered *Synechocystis* sp. PCC 6803 with a chromosomal integration of a heterologous mevalonate pathway has enabled production of isoprene (0.25 mg/g DW; Bentley *et al*., 2014; Lindberg *et al*., 2010). In addition, volatile ethylene has been produced from CO_2_ within recombinant *Synechocystis* sp. PCC 6803 strains (Shen and Liao, 2012). However, the low enzyme activity of isoprene synthase and the ethylene‐forming enzyme are still rate‐limiting. In addition to volatility, the target products have to be nontoxic to achieve continuous CO_2_ conversion. The production of isobutyraldehyde (less toxic) has been shown to be more suitable than isobutanol production in *Synechococcus elongatus* PCC 7942 (Lan and Liao, 2012). If the target product is highly toxic to cells, an in situ recovery process is required. Plant terpenoids such as limonene (4 mg/L) and α‐bisabolene (0.6 mg/L) have been synthetized by engineered *Synechococcus* sp. PCC 7002 in a nitrogen‐free medium (Davies *et al*., 2014). To reduce the toxicity of the products, a simple dodecane overlay was used for *in situ* extraction.

**Figure 1:**
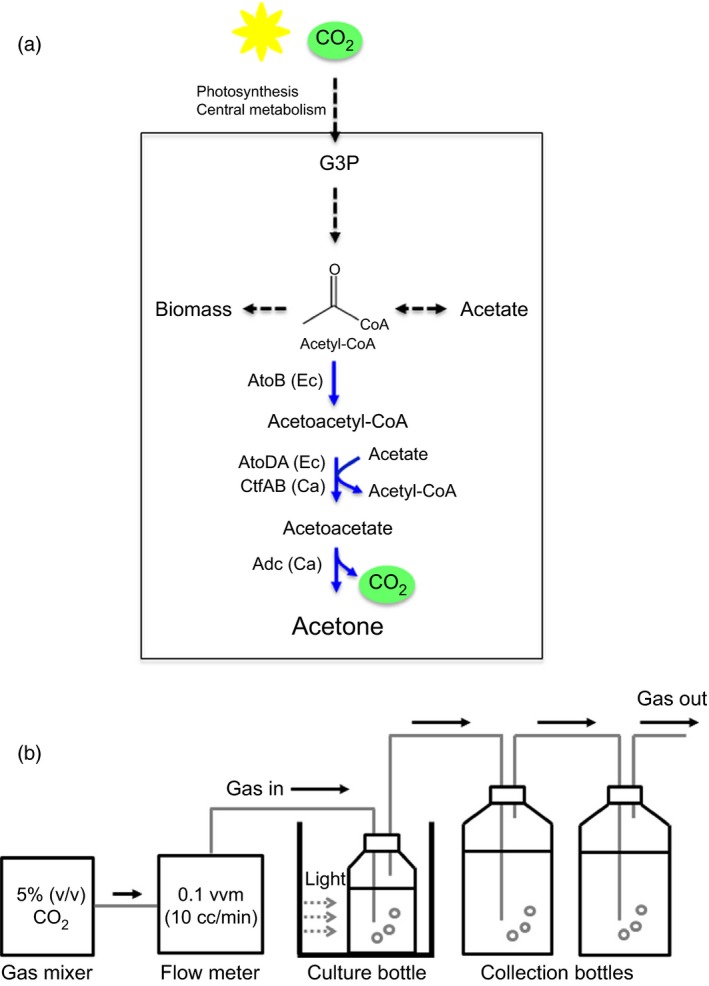
Cyanobacterial acetone production by introducing heterologous pathway. (a) A schematic pathway for acetone production in recombinant *Synechococcus elongatus* PCC 7942 strains (SeAAD and SeACD) expressing genes encoding for heterologous *Escherichia coli* AtoB thiolase, *E. coli* AtoDA or *Clostridium acetobutyricum* AtoDA acetoacetyl‐CoA acyltransferase, and *C. acetobutyricum* Adc acetoacetate decarboxylase. (b) Schematic diagram of the cyanobacterial culture bottle with CO_2_ bubbling and a gas‐stripping‐based recovery system: (a) Mixed gas of 5% (v/v) CO_2_ and 95% (v/v) air was continuously provided at 10 mL/min (0.1 vvm) into a culture bottle, and the off‐gas was diverted to two collection bottles (2 × 100 mL).

Acetone has a low boiling point and is a flammable chemical used extensively as a solvent in paints, coatings and adhesives or as an intermediate in the production of polymers. Regarding promising chemicals for continuous CO_2_ conversion, acetone is also a good candidate because it is relatively volatile and less toxic to cyanobacterial cells. To establish sustainable production of acetone using cyanobacteria, a synthetic pathway based on a *Clostridium* pathway (converting acetyl‐coA to acetone using acetyl‐CoA acetyltransferase, acetoacetyl‐CoA transferase and acetoacetate decarboxylase) has been designed and integrated to produce acetone in *Synechocystis* sp. PCC 6803 (Zhou *et al*., 2012; Figure 1a). After the engineered strains were cultivated under nutritional limitations (nitrogen and phosphorus free), metabolic shifting under light and aerobic condition to dark and anoxic condition achieved production of acetone (36 mg/L) and acetate (40 mg/L) in the medium in a sealed test tube. Similarly, *S. elongatus* PCC 7942 has been engineered for isopropanol production via the acetone‐producing *Clostridium* pathway combined with NADPH‐dependent secondary alcohol dehydrogenase activity (Kusakabe *et al*., 2013). The engineered *S. elongatus* strains have required metabolic shifting for the production of isopropanol (26.5 mg/L) and acetate (150 mg/L). Further optimizations by dark fermentation and anaerobic conditions to light and aerobic conditions have lead the enhanced production of isopropanol (146 mg/L; Hirokawa *et al*., 2015). However, none of strains has produced acetone under light and aerobic condition without a metabolic shift.

Herein, we report engineered *S. elongatus* PCC 7942 that produced sole acetone from continuous feeding of CO_2_ under light and aerobic conditions. The product was simultaneously separated from the culture medium. Photosynthetic acetone production was approached in a different way than in previous studies, by replenishing intracellular acetyl‐CoA directly from CO_2_ via ATP‐driven carbon flux and a phosphoketolase (PHK)‐phosphotransacetylase (Pta) pathway. Finally, we determined that synthetic pathway design for increase the acetyl‐coA supply is the most critical factor for real photosynthetic production of acetone and demonstrated a sustainable photosynthetic platform for the production of acetyl‐CoA‐derived biochemicals.

## Results and discussion

### Analysis of acetone‐producing *S. elongatus* PCC 7942 in the presence of acetate

**Figure 2:**
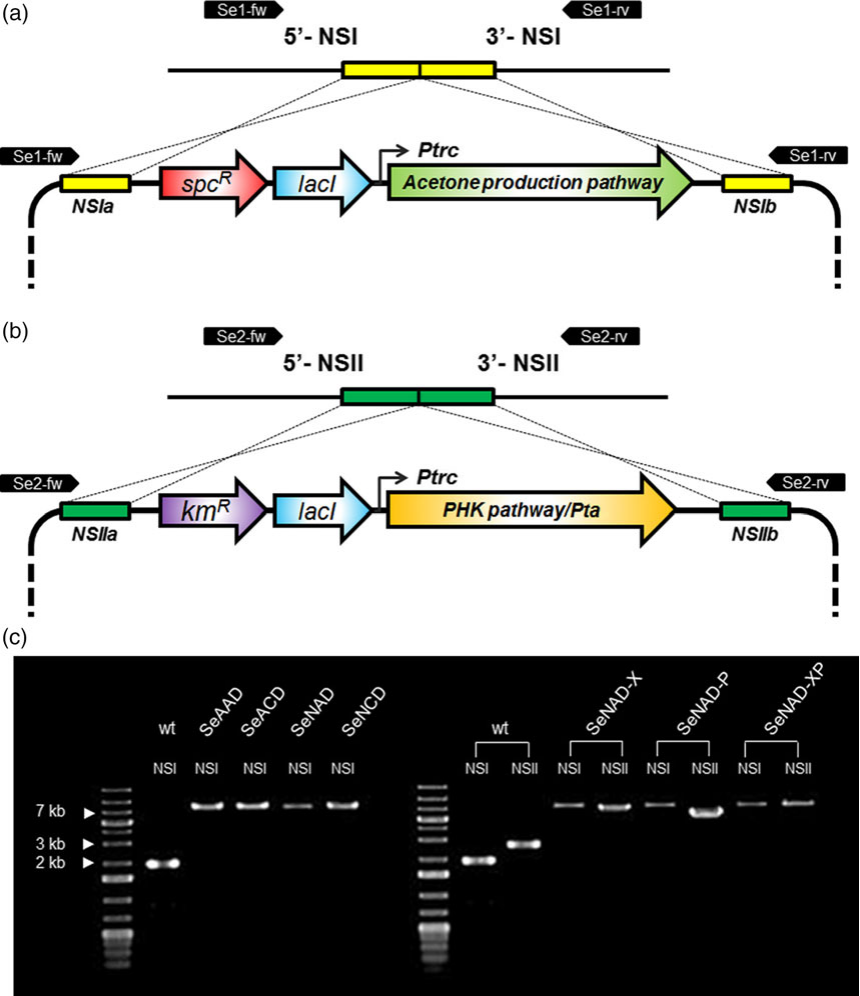
Scheme of heterologous gene integration into *Synechococcus elongatus* PCC 7942: (a) the acetone production pathway from acetyl‐CoA to acetone was integrated into NSI of the genome. (b) Genes encoding for phosphoketolase (PHK) or/and phosphotransacetylase (Pta) were integrated into NSII of the genome. (c) Colony PCR results verifying recombinant *S. elongatus* strains using a pair of Se1‐fw/rv and Se2‐fw/rv for the NSI and NSII integrations, respectively. The DNA sequences were also correctly verified. The target size of each PCR product for cyanobacterial wild type or mutant: wild type (1.9 kb), SeAAD (8.1 kb), SeACD (8.1 kb), SeNAD (7.9 kb), SeNCD (7.9 kb) at NSI and wild type (2.7 kb), SeNAD‐X (7.2 kb), SeNAD‐P (5.8 kb) and SeNAD‐XP (8.2 kb) at NSII.

**Figure 3:**
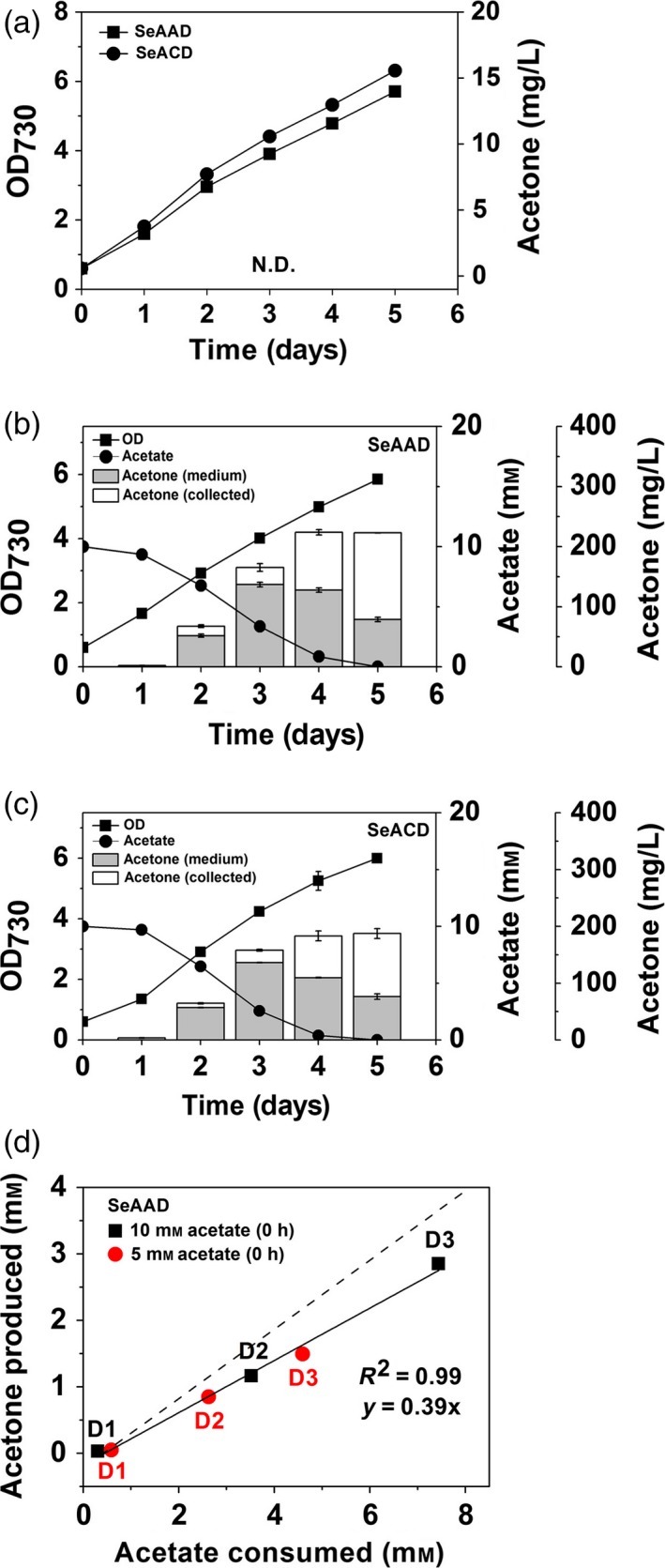
(a) Cyanobacterial growth (OD_730_) and acetone measurements of the SeAAD and SeACD strains in the absence of acetate under constant light and 5% CO_2_ feeding. The duration of cyanobacterial growth (OD_730_), and acetone production of the (b) SeAAD and (c) SeACD strains, in the presence of potassium acetate (10 mm) under constant light and 5% CO_2_ feeding. Acetone in the medium (grey) and in the collection bottle (white) was measured using the gas‐stripping‐based recovery systems. (d) Correlation of acetate consumed and acetone production by the SeAAD strain with either 5 mm (red) or 10 mm (black) potassium acetate at 0 h. D1, D2 and D3 represent Day 1, Day 2 and Day 3 after inoculation, respectively. The slope of the dashed line is a theoretic conversion yield (mol/mol) by 2 mole of acetone produced from 1 mole of acetate consumed. All data are mean ± SD from triplicate cultures. N.D., not detected.

The first generation strains of *S. elongatus* PCC 7942 used for photosynthetic acetone production were constructed (Table 1; Figure 2), resulting SeAAD (*S. elongatus* NSI::Bb1s‐*atoB*‐*atoDA*‐*adc*) and SeACD (*S. elongatus* NSI::Bb1s‐*atoB*‐*ctfAB*‐*adc*) expressing heterologous *Escherichia coli* and *Clostridium acetobutyricum* genes (Figure 1a). The strains showed no production of acetone under 5% CO_2_ bubbling in constant light as reported previously (Kusakabe *et al*., 2013; Figure 3a). Acetate can be used as a co‐substrate for acetoacetyl‐CoA transferase (AtoDA/CtfAB) or as a substrate to supply Acetyl‐CoA catalysed by Acetyl‐CoA synthetase (Synppc7942_1352) in the acetone production pathway. Thus, we added 10 mm of potassium acetate to the medium when cells were inoculated. As a result, 222.7 mg/L ± 0.17 and 187.3 mg/L ± 8.85 of total acetone (the sum of acetone measured in the medium and captured in the collection bottle) were produced by the SeAAD and SeACD strains, respectively, over 5 days in the presence of acetate (Figure 3b,c). The SeAAD strain having AtoD showed slightly higher production of acetone than did SeACD in the presence of acetate. Although the highest production level of acetone was reported in this study, we measured decreased levels of acetate in the medium. Both SeAAD and SeACD completely utilized 10 mm acetate in the medium within 5 days. When the acetate was completely depleted, production of acetone by cyanobacterial cells stopped.

**Table 1:** Bacteria strains and plasmids used in this study

Strain or plasmid	Relevant characteristics	Reference
Strains		
*Escherichia coli* HIT‐DH5α	F^−^ (80d *lac*Z M15) (*lac*ZYA‐*arg*F) U169 *hsd*R17(r^−^ m^+^) *rec*A1 *end*A1 *rel*A1 *deo*R96	RBC Bioscience
*Synechococcus elongatus* PCC 7942	Wild type (ATCC 33912)	ATCC
SeAAD	*S. elongatus* PCC 7942 NSI::Bb1s‐AAD	This study
SeACD	*S. elongatus* PCC 7942 NSI::Bb1s‐ACD	This study
SeNAD	*S. elongatus* PCC 7942 NSI::Bb1s‐NAD	This study
SeNCD	*S. elongatus* PCC 7942 NSI::Bb1s‐NCD	This study
SeAAD‐XP	*S. elongatus* PCC 7942 NSI::Bb1s‐AAD NSII::Bb2k‐XP	This study
SeACD‐XP	*S. elongatus* PCC 7942 NSI::Bb1s‐ACD NSII::Bb2k‐XP	This study
SeNAD‐X	*S. elongatus* PCC 7942 NSI::Bb1s‐NAD, NSII::Bb2k‐X	This study
SeNAD‐P	*S. elongatus* PCC 7942 NSI::Bb1s‐NAD, NSII::Bb2k‐P	This study
SeNAD‐XP	*S. elongatus* PCC 7942 NSI::Bb1s‐NAD NSII::Bb2k‐XP	This study
Plasmids		
pBbE1c‐GFP	ColE1, Cm^r^, P_*trc*_,	Lee *et al*. (2011)
pSyn_1	pUC, Spc^r^, P_*Ni*_, NSI target sites	Invitrogen
pSe1Bb1s‐GFP	pUC, Spc^r^, LacI, P_*trc*_, *gfp*, NSI target sites SyneBrick vector, a derivative of pBbE1c‐GFP and pSyn_1	This study
pSe2Bb1k‐GFP	pUC, Km^r^, LacI, P_*trc*_, *gfp*, NSII target sites SyneBrick vector, a derivative pSe1Bb1s‐GFP	This study
pSe1Bb1s‐AAD	pUC, Spc^r^, LacI, P_*trc*_, NSI target sites, *atoB*,* atoDA*,* adc* gene	This study
pSe1Bb1s‐ACD	pUC, Spc^r^, LacI, P_*trc*_, NSI target sites, *atoB*,* ctfAB*,* adc* gene	This study
pSe1Bb1s‐NAD	pUC, Spc^r^, LacI, P_*trc*_, NSI target sites, *nphT7*,* atoDA*,* adc* gene	This study
pSe1Bb1s‐NCD	pUC, Spc^r^, LacI, P_*trc*_, NSI target sites, *nphT7*,* ctfAB*,* adc* gene	This study
pSe2Bb1k‐X	pUC, Km^r^, LacI, P_*trc*_, NSII target sites, *xpkA* gene	This study
pSe2Bb1k‐P	pUC, Km^r^, LacI, P_*trc*_, NSII target sites, *pta* gene	This study
pSe2Bb1k‐XP	pUC, Km^r^, LacI, P_*trc*_, NSII target sites, *xpkA, pta* gene	This study

To investigate the correlation between acetate consumed and acetone produced, we analysed the acetone production by the SeAAD strain by providing different concentrations of acetate in the culture (5 and 10 mm) at 0 day. The correlation coefficient was significantly high (*R* ^2^ = 0.99) for acetate consumed with acetone produced (Figure 3d). When we calculated the ratio of acetate consumed and acetone produced, 0.39 (mole of acetone/mole of acetate) was calculated, where the theoretical conversion molar yield for the acetone from acetate is 0.5 (mole of acetone/mole of acetate). Therefore, a strong positive correlation between acetone production and acetate consumption was shown. This suggested that carbon flux from acetyl‐CoA to acetone was derived from the acetate given to the SeAAD strain. Thus, inefficient supply of acetyl‐CoA level could not drive the subsequent enzymatic reactions for acetone production under constant light and in aerobic culture, in the absence of acetate.

### ATP drives photosynthetic sole acetone production in the absence of acetate

**Figure 4:**
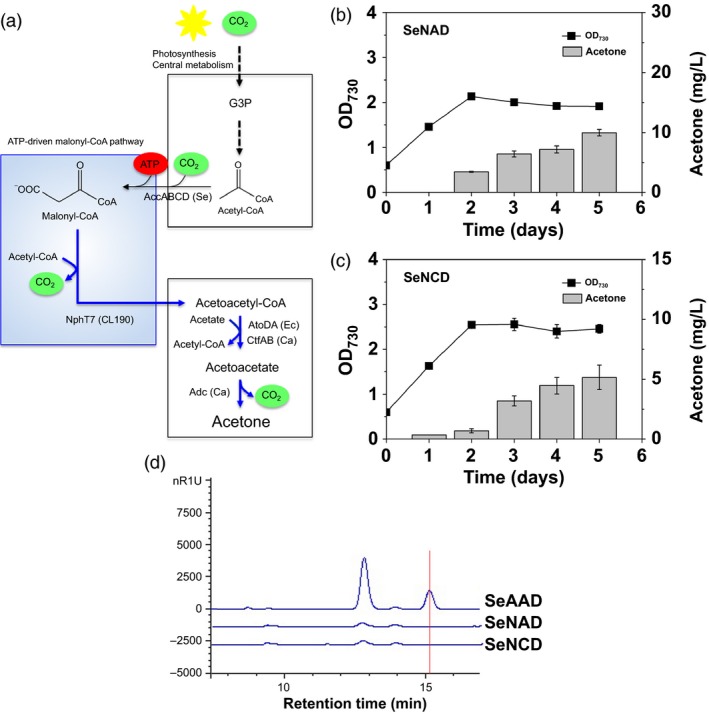
Photosynthetic sole acetone production via ATP‐driven malonyl‐CoA synthesis: (a) a scheme of acetone production via ATP‐driven malonyl‐CoA pathway. Cyanobacterial growth (OD_730_) and acetone measurements were measured in absence of acetate under the constant light and 5% CO_2_ feeding using the (b) SeNAD and (c) SeNCD strains. All data are mean ± SD from triplicate cultures. (d) The HPLC chromatograms were shown for the supernatants in the culture medium of each SeAAD, SeNAD and SeNCD strain. A peak of acetate was indicated at the retention time 15.1 min (red line).

The AtoB thiolase used in the SeAAD and SeACD strains favours the thiolysis of acetoacetyl‐coA. Recent studies have showed that ATP‐driven malonyl‐CoA synthesis and the decarboxylation reaction in engineered *S. elongatus* PCC 7942 enabled direct photosynthetic 1‐butanol production (29.9 mg/L; Lan and Liao, 2012; Lan *et al*., 2013). ATP‐driving metabolic force was capable of supplying acetoacetyl‐CoA from acetyl‐CoA. Thus, we constructed the two strains, SeNAD (*S. elongatus* NSI::Bb1s‐*nphT7*‐*atoDA*‐*adc*) and SeNCD (*S. elongatus* NSI::Bb1s‐*nphT7*‐*ctfAB*‐*adc*), for acetone production to having two‐step reactions catalysed by native AccABCD acetyl‐CoA carboxylase and heterologous NphT7 thiolase/acetoacetyl‐CoA synthase (Okamura *et al*., 2010; Figure 4a).

The engineered strains SeNAD and SeNCD were cultivated under constant light and in aerobic culture. As a result, indeed, the SeNAD and SeNCD strains produced 9.96 mg/L ± 0.54 and 5.16 mg/L ± 1.03 of acetone, respectively (Figure 4b,c). The acetone production of the SeNAD strain was twice as high as that of SeNCD. None of the engineered cyanobacterial strains have shown growth‐associated production of acetone under light and in aerobic culture (Hirokawa *et al*., 2015; Kusakabe *et al*., 2013; Zhou *et al*., 2012). This is the first report of direct conversion of CO_2_ to acetone under aerobic and lighted conditions.

Interestingly, the growth reduction in SeNAD (67%) and SeNCD (59%) was also reflected in the growth of SeAAD and SeNAD, although the levels of acetone produced (10 mg/L) by the strain SeNAD were significantly lower than the cytotoxic level (between 10 and 100 g/L; Kusakabe *et al*., 2013). Introducing energy‐consuming ATP‐driven pathway could be cellular burden in cells. In addition, we checked whether metabolites other than acetone were produced or not, using HPLC and GC‐MS analysis. No acetate was measured in the supernatant of the SeNAD and SeNCD cultures (Figure 4d). Thus, our engineered SeNAD and SeNCD strains were capable of producing sole acetone from CO_2_ via NphT7‐mediated pathway. Furthermore, heterologous expression of acetate‐independent acetoacetyl‐coA transferase could be advantageous with the NphT7‐mediated pathway in no acetate given system (Alonso‐Gutierrez *et al*., 2013). Although the resulting strain produced sole acetone in lighted, aerobic culture, the levels of sole acetone were quite low due to the limited acetyl‐CoA pool.

### Increased acetyl‐CoA‐pool enhanced acetone production via the phosphoketolase pathway

**Figure 5:**
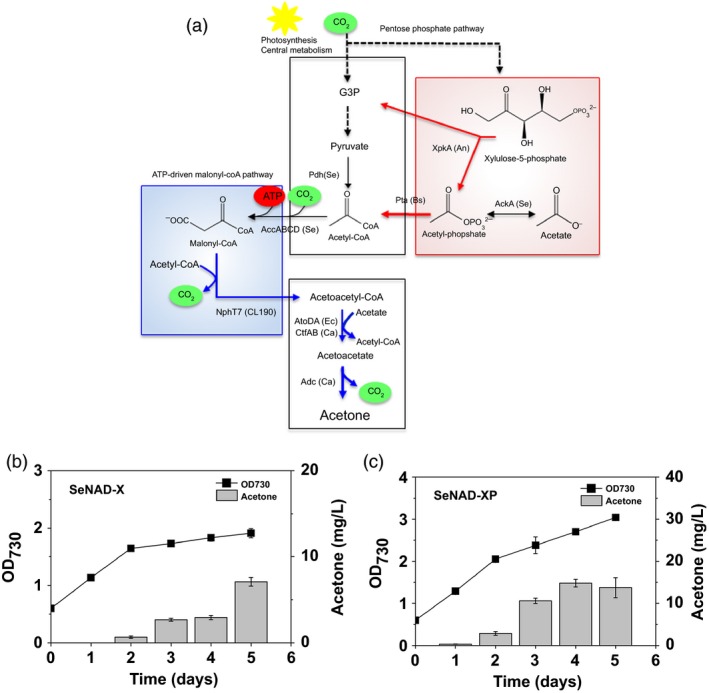
Photosynthetic sole acetone production via the phosphoketolase pathway: (a) a scheme of redirection of carbon flux from xylulose 5‐phosphate to the acetone production pathway via the phosphoketolase (PHK) pathway with phosphotransacetylase (Pta). Cyanobacterial growth (OD_730_) and acetone levels were measured in absence of acetate under constant light and 5% CO_2_ feeding using the (b) SeNAD‐X and (c) SeNAD‐XP strains. All data are mean ± SD from triplicate cultures.

To enhance acetone production via supplying the acetyl‐CoA, the SeNAD strain (best producer from the previous result) was chosen for further engineering with the phosphoketolase pathway (Figure 5a). The phosphoketolase pathway from *Aspergillus nidulans* (Papini *et al*., 2012) has contributed to increased levels of polyhydroxybutyrate (Kocharin *et al*., 2013) and fatty acid ethyl ester (de Jong *et al*., 2014) by supplying endogenous acetate and acetyl‐CoA, respectively. Thus, we constructed a SeNAD‐X (*S. elongatus* NSI::Bb1s‐*nphT7*‐*atoDA*‐*adc* NSII::Bb1s‐*xpkA*) strain that expressed the *A. nidulans xpkA* gene encoding for a phosphoketolase.

As a result, the SeNAD‐X strain produced 7.09 mg/L ± 0.51 acetone, which was 26% lower than the level from SeNAD (Figure 5b). As the PHK pathway has been reported for sugar dissimilation on xylose by some bacteria (bifidobacteria), xylose‐fermenting yeasts or filamentous fungi, those strains generate acetyl‐CoA from acetyl phosphate via either acetate kinase and acetyl‐CoA synthetase or Pta. Due to the lack of gene annotation of Pta in *S. elongatus* PCC 7942, the enhancement of acetone by redirecting carbon flux from xylulose‐5‐phosphate to acetyl‐CoA could be failed. This could explain that acetyl phosphate could be accumulated in the cells, SeNAD‐X. Accumulated acetyl phosphate could lead to a lower growth rate of SeNAD‐X than SeNAD due to altered status of lysine acetylation, which is related to post‐translational protein modification of a critical regulatory role (Mo *et al*., 2015; Weinert *et al*., 2013).

Thus, we co‐expressed the *Bacillus subtilis pta* gene encoding for phosphotransacetylase, yielding a NAD‐XP (*S. elongatus* NSI::Bb1s‐*nphT7*‐*atoDA*‐*adc* NSII::Bb1s‐*xpkA‐pta*) strain to completely metabolize xylulose‐5‐phosphate to acetyl‐CoA. As a result, the SeNAD‐XP strain produced 13.74 mg/L ± 2.39 of acetone (a 1.4‐fold increase), compared to the SeNAD strain, in the absence of acetate under aerobic and lighted conditions (Figure 5c). Also, no organic acids, including acetate, were measured in the medium after cultivation of SeNAD‐XP (data not shown). Interestingly, the SeNAD‐P (*S. elongatus* NSI::Bb1s‐*nphT7*‐*atoDA*‐*adc* NSII::Bb1s‐*pta*) strain showed no growth without direct evidences. The sole expression of Pta in the SeNAD‐P strain led to detrimental cell growth in the absence of acetate, yielding a possibly futile cycle of acetyl‐CoA via acetyl phosphate and acetate. However, their growth recovered in the presence of 10 mm acetate at 0 day. This rescue could be due to high flux of acetate conversion to acetyl‐CoA above the critical levels of acetyl‐CoA in a cell. Nonetheless, heterologous gene expression of Pta with the PHK pathway was necessary for cyanobacterial cells to enhance the photosynthetic production of acetone.

**Figure 6:**
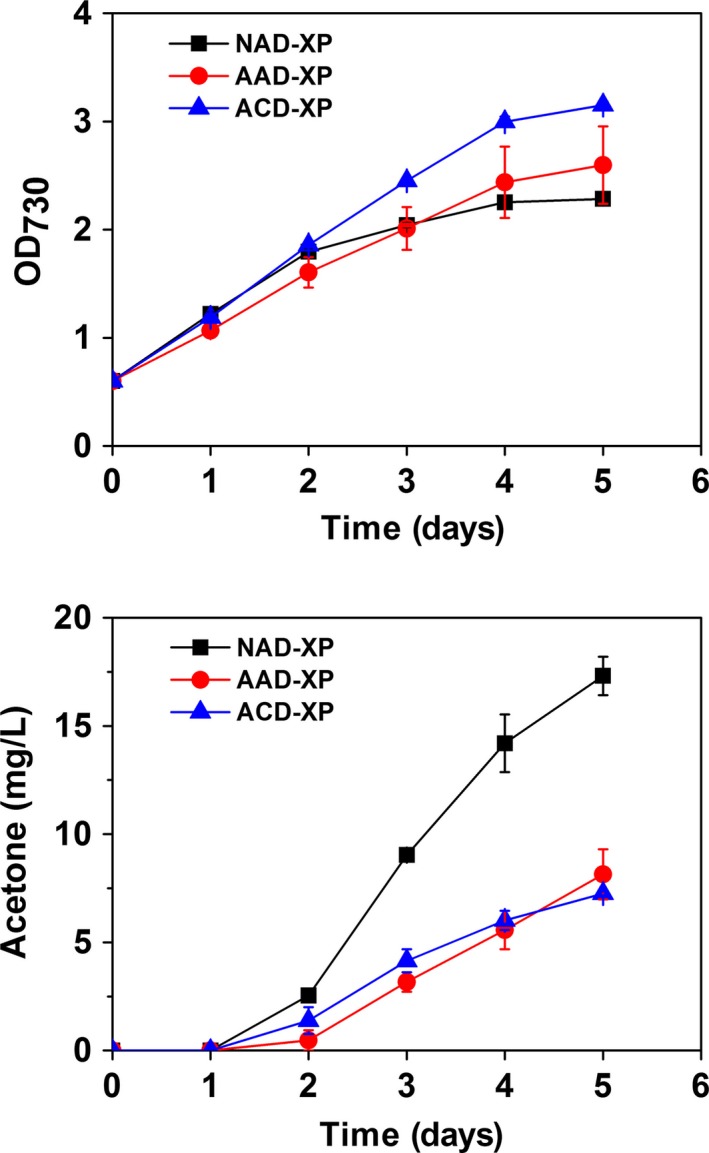
Trade‐off in the cell growth and acetone production in engineered cyanobacteria. Cyanobacterial growth (OD_730_) and acetone levels were measured in the absence of acetate under constant light and 5% CO_2_ feeding using the strains SeAAD‐XP (red circle), SeACD‐XP (blue triangle) and SeNAD‐XP (black square). All data are mean ± SD from triplicate cultures.

To investigate the cellular burden due to excessive consumption of ATP driven by NphT7 with xylulose‐5‐phosphate by PHK pathway for acetone production, the growth of the best producer SeNAD‐XP was compared with the growth of the strain SeAAD‐XP and SeACD‐XP strains where NphT7 was replaced with AtoB. As a result, the SeAAD‐XP and SeACD‐XP strains showed the higher cell growth than the strain SeNAD‐XP, but the acetone titres from both strains were significantly decreased (Figure 6). As discussed in previous studies (Lan and Liao, 2012; Lan *et al*., 2013), additional ATP consumption caused adverse effects in the acetone‐producing cells, resulting in reduced biomass formation. However, sole synthetic PHK pathway was not enough to provide the driving forces of carbon flux towards higher acetone production. The trade‐off in the cell growth and acetone production using the strain SeNAD‐XP could be achieved by optimizing expression of NphT7 using tight gene expression systems.

Supplying the acetyl‐CoA via the XpkA‐Pta pathway and incorporating an ATP dependent step contributed to increasing photosynthetic acetone production (13.74 mg/L ± 2.39) in *S. elongatus* PCC 7942 under aerobic and lighted conditions. Direct measurement of intracellular acetyl‐CoA could be useful to confirm the altered levels of acetyl‐CoA in the acetone‐producing cyanobacterial strains. Furthermore, a synthetic nonoxidative glycolysis pathway (Bogorad *et al*., 2013) using another PHK pathway (converting fructose 6‐phosphate to erythrose 4‐phosphate and acetyl phosphate) could be useful to analyse acetyl‐CoA metabolism in cyanobacteria. It might also be applied for the improvement of acetone and acetyl‐CoA‐derived chemicals.

### Continuous conversion of CO_2_ to sole acetone in a controlled flat photobioreactor

**Figure 7:**
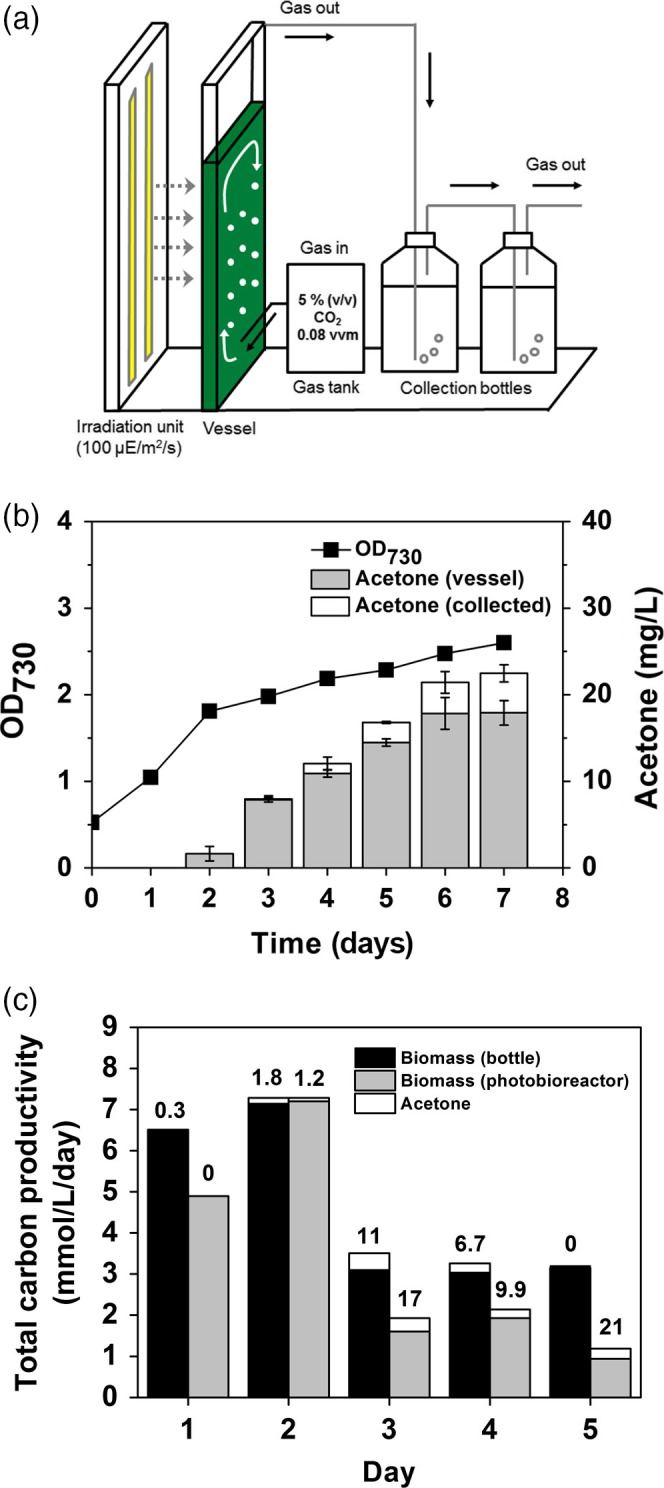
Conversion of CO_2_ to sole acetone in a controlled flat photobioreactor: (a) A schematic diagram of the cyanobacterial culture in a controlled flat photobioreactor with 5% (v/v) CO_2_ bubbling and a gas‐stripping‐based recovery system (two 500‐mL collection bottles; distilled water). (b) Cyanobacterial growth (OD_730_) under constant light and 5% CO_2_ feeding using the SeNAD‐XP strains was monitored. Acetone levels were measured in the bioreactor vessel and in the collection bottles. All data are mean ± SD from triplicate cultures. (c) Total carbon productivities (mmol/L/day) of the SeNAD‐XP strain were calculated from acetone production (white bar) and biomass when the cyanobacterial cells were cultivated either in the bottle (100 mL; black bar) or in the controlled vessel (1.8 L) of the photobioreactor (grey bar). The numbers above bars showed per cent carbon partitioned to acetone.

To increase production of photosynthetic acetone by SeNAD‐XP, the strain was cultivated in a controlled flat photobioreactor (1.8 L) with continuous 5% CO_2_ bubbling and constant light conditions (Figure 7a). Using the engineered SeNAD‐XP strain, a total of 22.48 mg/L ± 1.00 of acetone was secreted into the vessel and captured in the collection bottle (Figure 7b). The specific production of acetone in the photobioreactor (1.8 L) was 8.65 mg/L/OD_730,_ and it was increased by 1.9‐fold, compared to the specific production in a bottle (100 mL), but a lower growth rate was observed.

The total carbon productivity and carbon partitioning to acetone of the SeNAD‐XP strain were calculated. As a result, carbon partitioning to acetone of the SeNAD‐XP strain was different from the cyanobacterial culture systems, showing significantly higher carbon partitioning to acetone (21%) in the photobioreactor (Figure 7c). Because there were negative correlations of biomass formation with acetone production, optimization of the cyanobacterial cell culture systems would be necessary with long‐term cultivation to increase the productivity level of sole production of acetone. Moreover, development of a recycling system of CO_2_ in the off‐gas would be necessary to achieve 100% conversion of CO_2_, although this also depends on the size of the photobioreactors used.

## Conclusions

**Figure 8:**
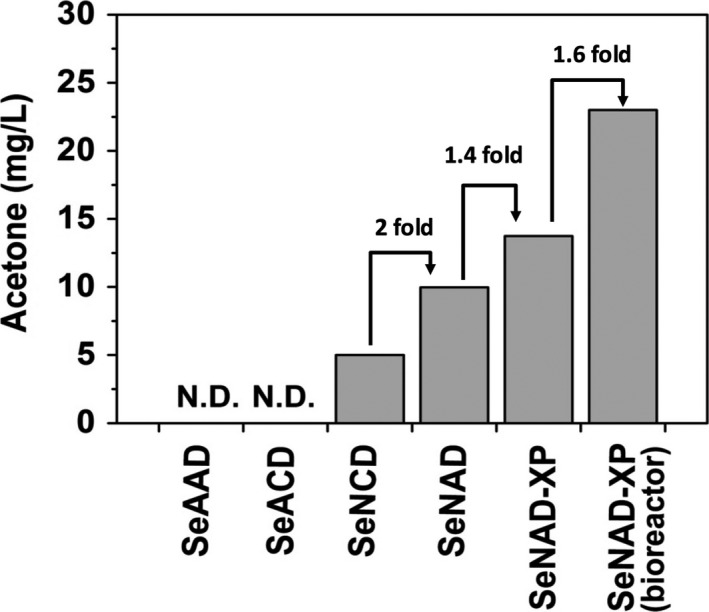
A summary of photosynthetic acetone productions from CO_2_ in metabolically engineered *Synechococcus elongatus* strains under constant light condition. None of acetone was produced in the strains SeAAD and SeACD. When NphT7 was replaced with A to B, acetone was produced for the first time under light condition in the SeNAD. In addition, phosphoketolase (PHK) pathway improved the acetone production in the SeNAD‐XP. The production titre was increased by optimizing culture vessel in a photobioreactor.

Acetyl‐coA, a key molecule for biotechnological applications in microbial metabolism, is used as a substrate for the oxidative TCA cycle, acetate, and ethanol production pathways, glyoxylate shunt or fatty acid biosynthesis (Choi and Lee, 2013; Steen *et al*., 2010). These acetyl‐CoA‐consuming pathways compete against the heterologous acetone‐producing pathway in *S. elongatus*. In this study, metabolically engineered *S. elongatus* PCC 7942 was successfully applied to continuously convert CO_2_ to sole acetone in lighted, aerobic culture (Figure 8). Rewiring the metabolisms with the PHK pathway replenished the pool of acetyl‐CoA that was converted to sole acetone along with the ATP‐driven carbon flux. Also, combining this with development of the cyanobacterial strain, bioprocessing for acetone recovery enhanced the production of acetone based on the principle of volatile gas–liquid exchange in a controlled photobioreactor. Thus, improvement of the cyanobacterial strains, combined with bioprocessing, will accelerate the development of biosolar cell factories to harvest volatile acetyl‐coA‐derived chemicals from CO_2_.

## Experimental procedures

### Strains and plasmids

All bacterial strains and plasmids used in this work are listed in Table 1. For cloning, *E. coli* strains were grown in Lysogeny broth medium (containing per litre: 10 g tryptone, 5 g yeast extract, and 10 g NaCl) at 37 °C, when appropriate, the medium was supplemented with 50 μg/mL kanamycin, 100 μg/mL spectinomycin. *S. elongatus* PCC 7942 was purchased from the ATCC 33912 and used as the production host for acetone from 5% (v/v) CO_2_ feeding. The neutral site I (NSI) or II (NSII) targeting vectors (SyneBrick vectors) were constructed by inserting either NSI DNA sequence or NSII DNA sequence from pSyn_1 into the BglBrick vectors (Lee *et al*., 2011) as a synthetic platform for gene expression in *S. elongatus* PCC 7942. SyneBrick vectors follow the strategy of the BglBrick cloning method to clone target genes into the vectors at the BglBrick restriction enzymes site (*Eco*RI, *Bgl*II, *Bam*HI and *Xho*I). The *E. coli atoB* (Hanai *et al*., 2007) and *atoDA* (Hanai *et al*., 2007)*, C. acetobutyricum ctfAB* (Hanai *et al*., 2007; Zhou *et al*., 2012) and *adc* (Hanai *et al*., 2007; Zhou *et al*., 2012)*, Streptomyces* sp. *CL190 nphT7* (Lan and Liao, 2012; Okamura *et al*., 2010), *Aspargillus nidulans xpkA* (Panagiotou *et al*., 2008; Zhou *et al*., 2012) *and Bacillus subtilis pta* (Zhou *et al*., 2012) genes were codon‐optimized using Gene Designer 2.0 software (DNA2.0; Menlo Park, CA) and synthesized (Genscript Inc., Piscataway, NJ) for efficient heterologous expression in *S. elongatus* PCC7942. Each target gene for acetone production was cloned into SyneBrick vectors (targeting at NSI) to construct a pSe1Bb1s‐AAD and pSe1Bb1s‐ACD. The *atoB* gene in the pSe1Bb1s‐AAD and pSe1Bb1s‐ACD was replaced to the *nphT7* gene, yielding a pSe1Bb1s‐NAD and pSe1Bb1s‐NCD, respectively. To enhance the acetyl‐CoA availability, additional *xpkA* or/and *pta* genes were cloned into SyneBrick vectors (targeting at NSII), yielding pSe2Bb1k‐X, pSe2Bb1k‐P and pSe2Bb1k‐XP.

### Transformation of *S. elongatus*

Transformation of *S. elongatus* PCC 7942 was performed as described previously (Golden *et al*., 1987). The SyneBrick vectors were transferred for chromosomal integration. Recombinant strains were obtained after transferring colonies to fresh selective plates in order to prevent from chromosome segregation. The strains were confirmed by PCR to verify integration of targets into the chromosome (Figure 2), and the DNA sequences were also correctly verified using a pair of Se1‐fw (5′‐TCT ACT ACA TCT GCC AAC CCA G‐3′) and Se‐1‐rv (5′‐AAT CTG AAG ACC CGC CAA CTG T‐3′) for the NSI and a pair of Se2‐fw (5′‐ATT GTT GAG GCA GGC AAT CAC G‐3′) and Se2‐rv (5′‐TGT CTA CAG CAC AGA CCA ATG G‐3′) for the NSII.

### Cyanobacterial culture for acetone production

*Synechococcus elongatus* PCC 7942 and its derivatives (Table 1) were cultivated at 30 °C in the 100 mL culture (Duran bottle with a three‐ports cap) under continuous fluorescent light (100 μE/m^2^/s) measured by LightScout Quantum meter (3415FXSE; Spectrum, Aurora, IL) in BG‐11 medium supplanted with 10 mm MOPS. 5% (v/v) CO_2_ gas (monitored by online gas analyser) and 95% (v/v) filtered air were supplied at constant flow rate of 10 cc/min into the medium (Figure 1b). Off‐gas line was sequentially connected to two identical collection bottles (500 mL), which contain 500 mL distilled water without headspaces. To collect volatile acetone from either the BG‐11 or the off‐gas line, a gas‐stripping‐based recovery systems (Inokuma *et al*., 2010) were modified (Figure 1b). Ten micrograms per milliliter spectinomycin or/and 10 μg/mL kanamycin were supplemented for selection pressure. One millimoar IPTG was supplemented into the culture medium at 24 h after inoculation.

### Quantification of acetate, alcohols and acetone

One milliliter of cyanobacterial cell supernatant or sample from collection bottle was filtered with syringe filter (pore size of 0.2 μm) after centrifugation at 10 000 ***g*** for 10 min. For quantification of acetone, ethanol and isopropanol, the samples were analysed by gas chromatography (Model 6890; Agilent Technologies, Santa Clara, CA) equipped with a HP‐INNOWAX polyethylene glycol column (30 m × 0.25 mm × 0.25 m) and a flame ionization detector (FID) under the following conditions: oven temperature, from 50 to 240 °C at a rate of 10 °C/min; injector temperature, 250 °C; detector temperature, 250 °C; carrier gas (He); flow rate, 25 mL/min; and split ratio of 1 : 10. For the measurement of the acetate, culture supernatant was passed through a syringe filter after centrifugation at 10 000 ***g*** for 10 min. The concentrations of acetate were determined by high‐performance liquid chromatography (HPLC system Agilent 1260; Waldbronn, Germany) equipped with a refractive index detector (RID) and an Aminex HPX‐87 H Ion Exclusion Column (300 mm by 7.8 mm; Bio‐Rad, Hercules, CA) under the following conditions: sample volume of 20 μL, mobile phase of 5 mm H_2_SO_4_, flow rate of 0.6 mL/min and column temperature of 65 °C.

### Controlled cyanobacterial culture in a flat‐panel photobioreactor

For controlled cyanobacterial culture, a flat‐panel photobioreactor (Labfors 5 Lux‐LED flat‐panel option [637 mm (L) × 298 mm (W) × 79 mm (D); INFORS‐HT, Bottmingen, Switzerland] was used to improve production of acetone. Cyanobacterial cells in exponential phase were centrifuged and diluted to OD_730_ of 0.5 in 1.8 L of BG‐11 medium. The flat‐panel vessel was illuminated under condition of continuous light (100 μE/m^2^/s), and 30 °C. 5% CO_2_ (v/v) was supplied into the cell medium at flow rate of 140 mL/min (0.08 vvm). 3 N NaOH was used to adjust pH 7 in BG‐11. Off‐gas line was connected to two 500‐mL collection bottles that contain 500 mL distilled water for the measurement of total acetone production. For the production of acetone, 1 mm IPTG was added at 24 h after inoculation.

### Calculations for total carbon productivity

Total carbon productivity was calculated from the previous calculations (Oliver and Atsumi, 2015). Cyanobacterial biomass was calculated based on the measurement of optical density (the conversion factor, 0.22 g DW/L/OD_730_). The carbon mole was calculated from the carbon contents (51.34% of cell biomass using the elemental composition) in a period, divided by 12 g/mole of carbon (Shastri and Morgan, 2005). To calculate total carbon productivity, carbon in acetone (mmol/L) was also calculated from the concentration of total acetone in a period by multiplied by 3 (carbon mole of Acetone C_3_H_6_O/ per CO_2_ fixation). Partitioning of acetone is calculated by carbon productivity of acetone dived by total carbon productivity.
